# Teaching health science students foundation motivational interviewing skills: use of motivational interviewing treatment integrity and self-reflection to approach transformative learning

**DOI:** 10.1186/s12909-015-0512-1

**Published:** 2015-12-21

**Authors:** Schoo A. M., Lawn S., Rudnik E., Litt J. C.

**Affiliations:** Professor, Rural Clinical School, Flinders University, PO Box 3570, Mount Gambier, 5290, South Australia Australia; Professor, Department of Psychiatry, Flinders Human Behaviour and Health Research Unit, Flinders University, Room 4T306 Margaret Tobin Centre, PO Box 2100, Adelaide, South Australia 5001 Australia; Senior Lecturer, Rural Clinical School, Flinders University, PO Box 889, Nuriootpa, SA 5355 Australia; Associate Professor, Discipline of General Practice, Flinders University, Bedford Park, South Australia Australia

**Keywords:** Motivational interviewing, Clinical education, Self-reflection, Transformative learning

## Abstract

**Background:**

Many undergraduate and graduate-entry health science curricula have incorporated training in motivational interviewing (MI). However, to effectively teach skills that will remain with students after they graduate is challenging. The aims of this study were to find out self-assessed MI skills of health students and whether reflecting on the results can promote transformative learning.

**Methods:**

Thirty-six Australian occupational therapy and physiotherapy students were taught the principles of MI, asked to conduct a motivational interview, transcribe it, self-rate it using the Motivational Interviewing Treatment Integrity (MITI) tool and reflect on the experience. Student MI skills were measured using the reported MITI subscores. Student assignments and a focus group discussion were analysed to explore the student experience using the MITI tool and self-reflection to improve their understanding of MI principles.

**Results:**

Students found MI challenging, although identified the MITI tool as useful for promoting self-reflection and to isolate MI skills. Students self-assessed their MI skills as competent and higher than scores expected from beginners.

**Conclusions:**

The results inform educational programs on how MI skills can be developed for health professional students and can result in transformative learning. Students may over-state their MI skills and strategies to reduce this, including peer review, are discussed. Structured self-reflection, using tools such as the MITI can promote awareness of MI skills and compliment didactic teaching methods.

**Electronic supplementary material:**

The online version of this article (doi:10.1186/s12909-015-0512-1) contains supplementary material, which is available to authorized users.

## Background

### Motivational interviewing in chronic disease management

Motivational interviewing (MI) is a collaborative, person-centred form of guiding conversation undertaken by health professionals to elicit and strengthen clients’ motivation to change [1]. It is internationally recognised as an effective intervention for supporting people to make positive changes in the management of their chronic conditions and associated lifestyle behaviours, and reducing the risk of further health comorbidity [2–7]. When MI skills are incorporated into training, health students report greater confidence in their ability to support Chronic Condition Self-Management (CCSM) and display improved knowledge and skills required to do so [8–10]. This is important since, in many countries, chronic disease burden is increasing and reducing this burden is a priority [11, 12].

**Fig. 1:**
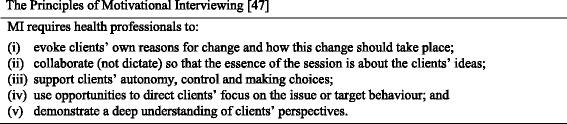
The Principles of Motivational Interviewing [47]

Effective ways to tackle chronic conditions include supporting behaviour change and encouraging self-management of the client as part of core health professional practice [13]. Consequently, health students need to develop the knowledge and skills required to deliver effective CCSM support [14–16]. This has implications across all health profession educational programs. However, research consistently shows that students are ill-prepared for supporting clients in behaviour change, which impacts on their clinical placements and practice once they graduate and become health professionals [8, 9, 17]. Therefore, understanding the principles of MI (Fig. 1) and maximising training opportunities are critical.

Training health professionals to be competent in MI skills requires sufficient instruction, opportunity for practice and reflection, provision of feedback, and ongoing follow up [18–22]. MI skills can be difficult to acquire and there is growing evidence that the spirit of MI (i.e., evocation, collaboration and supporting autonomy) is more important than the technical skills [1, 23–25]. Attitudes held by the health professional and the ability to reflect are important since behavioural change needs to be elicited rather than imposed. It requires understanding, flexibility and skilful guiding in response clients’ needs [26].

MI differs substantially from other more general interviewing techniques that enhance therapeutic alliance with clients. It relies on relational and unique technical components involving differential evocation and reinforcement of client change [25]. It requires the interviewer to evoke, collaborate and support autonomy whilst showing empathy and providing direction to the interview; a complex task. For a health practitioner to be competent in MI they must value and practice the process of consultation and reflection to understand a client’s perspective [27]. Reflection is a form of communication that involves dialogue with self and/or others that can produce an altered perspective [28]. Reflection has been identified as a crucial element for students to achieve ‘transformative learning’ and, as argued by Mezirow [29], has become influential in adult education [30]. Transformative learners are reflective and more likely to respectfully consider alternate opinions and integrate new ideas within their professional practice [28]. Therefore, these are important capabilities that educators need to facilitate in students who wish to practice MI. It is thus posited that students who have the opportunity to participate in self-reflective learning activities may achieve transformative learning and improve their readiness to practice MI. Although reflective practitioners are likely to learn from their clients and improve over time [26], MI is perceived as difficult to learn [31] and research that considers the value of self-reflection for MI training is needed [32, 33].

### Reflective learning

Preparing the future health workforce for clinical practice and utilising interprofessional education (IPE) to develop competence are important issues [34]. The University’s curriculum for occupational therapy (OT) and physiotherapy (PT), aims to develop students’ competence through reflective learning and practice [35]. Self-assessment, as part of this reflection, helps to facilitate deep learning [36, 37] where students become reflective practitioners who enhance and maintain competence after graduation [38]. Reflection represents the highest skill level in the Structure of the Observed Learning Outcome (SOLO) taxonomy [35], and is captured in the literature by terms such as ‘critical reflection’, ‘reflective learning’, ‘reflective thinking’, ‘metacognitive reflection’, ‘mindfulness’, ‘critical thinking’ and ‘reflective judgement’ [39, 40]. Reflective thinking is transformative [35] and connects theory and practice [41]. It can enhance practitioners’ responses to clients in unpredictable situations [42].

Reflective thinking, assessment and learning are action research-based (reflect-plan-act-observe-reflect etc.) and are part of a cyclic process that enables students and health professionals to become better practitioners through lifelong learning [43]. Transformative learning occurs when a student is presented with information, experiences that challenge and alters attitudes, values and behaviours [44]. Reflective practice is a core skill that equips graduates for transformative learning and client-centred care. This can be difficult when students are educated in a more task-orientated environment where professionals prescribe solutions to clients to treat their diseases. There is also considerable evidence that health professionals frequently revert back to their old practices over time of directing and prescribing [45–50] instead of negotiating [51].

Given these considerations, the two key questions were: (i) What are the self-assessed MI skills of pre-registration health students who have participated in a program of one didactic MI lecture, two practical tutorials and one simulated interview?; and (ii) Does a structured self-reflective task using the Motivational Interviewing Treatment Integrity (MITI) tool promote transformative learning?

## Methods

### Design

This study used a mixed methods design with a qualitative content analysis of a student focus group and excerpts from reflective assignments used to answer the first research question. Quantitative assessment of students MITI scores (Global Spirit Rating, Evocation, Collaboration, Autonomy, Direction, Empathy) were used to answer the second research question.

### Participants

Participants were all 36 students, Master of Occupational Therapy (OT) (*n* = 17, 14 female) and Master of Physiotherapy (PT) students (*n* = 19, 11 female) enrolled in an interprofessional practice core topic as part of their first year in a 2-year graduate-entry program at an Australian university to introduce concepts such as collaborative practice, primary health, health promotion, chronic disease management and case management. Of the 36 students who were required to submit a reflective assignment, 22 reported all MITI results (*n* = 22, 15 female, 13 PT). Following the submission of their required MI assignment, one focus group was conducted involving four PT students (two females) and one OT student (female) who volunteered to provide further group reflection on the process. Students were aware that the focus group was part of a research project and that participation was voluntary. All participants provided written consent.

### Teaching procedure

Following one topic lecture on the essence of MI and two practical tutorials where students from the two disciplines practiced in mixed groups of three (interviewer, interviewer and observer) with support of a tutor, students were required to apply their learnings by conducting a motivational interview with a family member or friend involving physical activity and/or exercise. They were also provided with the MITI tool, sample questions and a decisional balance list to assist practicing the interview process and rating it. Students were required to demonstrate insight in the application of key principles of MI (i.e., evocation, collaboration, supporting autonomy, directing and empathy, and asking open-ended questions), to facilitate the person’s ownership of process goals. Students were to help clients focus on the process to achieve results rather than solely on the desired outcome to enhance their health and wellbeing, and deal with any enablers and/or barriers.

Students were free to choose for their dialogue the type of ‘client’ as well as one of three behaviours they wished to facilitate changing (i.e. enhancing physical activity and/or exercise, or wearing an orthotic or prosthetic device). Fidelity of the process was enhanced by students audio-recording the interviews and transcribing and rating them using the Motivational Interviewing Treatment Integrity (MITI) tool [52] and students were encouraged to practice coding transcripts. Aiming for competence in conducting high fidelity simulated interviews to promote physical activity and exercise is important since it enhances the effect of the intervention [53]. The focus of this interview and the required reflective assignment was upon the use and identification of MI skills (i.e., reflecting on ‘what could you have done differently’) rather than the content or topic of the interview. The engagement of a ‘client’ known by or related to the student was not deemed problematic for both, the client and the interviewer, due to their relationship and the attention by the latter on learning to follow a process.

Students were provided with guidelines on writing the reflective assignments (Additional file 1), together with the marking criteria (Additional file 2). They submitted their recordings and transcripts together with their assignments reflecting on their experiences, self-assessed MITI scores and quality of their interviews, and what they perceived they could have done differently.

### Focus group session

**Fig. 2:**
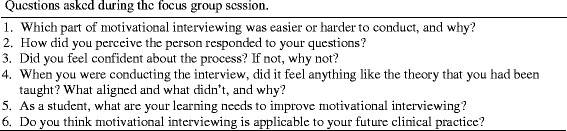
Questions asked during the focus group session

Following the lecture and tutorials, a focus group was conducted with those students who indicated interest in participating. The session was run by the course coordinator who was not personally involved in the lectures or tutorials, and students were encouraged to provide feedback on how they found the process of undertaking, recording and transcribing the interviews and critically self-evaluating their performance. Figure 2 shows the six questions asked during the focus group session that were made available to the students before the session to maximise their potential for reflective input.

### Outcome measures

The MITI tool is the benchmark for assessing MI treatment integrity [54, 55] and considered less complicated than its parent instrument the Motivational Interviewing Skill Coded (MISC) since it does not require rating the client’s behaviour during the interview [56]. As the focus of this study was upon the behaviour of the student rather than the client, the MITI was selected. The MITI is likely to be more useful to describe the degree to which the intervention/dialogue reflects the paradigms of MI. It is also useful for providing targeted feedback on areas in which the interviewer is performing well, and how they can improve [52]. The MITI tool measures the interviewer’s use of evocation, collaboration, supporting autonomy, direction and empathy as well as their percentage use of motivation adherent questions, open-ended questions and questions that require simple or complex reflection. Recorded interview segments of 10 min were found to yield comparable reliability and integrity results when compared with 20 min segments [56]. The MITI is suitable for both novice and experienced clinician use. It has global ratings for each of the five MI key principals, and six behaviour counts for MI adherent versus non-adherent questions, open versus closes questions and simple versus complex reflections. Reliability estimates for items ranges between fair and excellent with some rater experience variation [55]. Therefore, for our study, 10 min interviews were deemed appropriate in length for students. Also, in line with recommendations by Moyers and colleagues [52], students were taught in class how to code their interview and advised to practice coding to enhance reliability and to start with coding Level I competencies (parsing utterances (defined as a completed thought or idea), giving information and open/closed questions) before coding Level II competencies (adding reflections, and asking MI adherent and non-adherent questions) and Level III competencies (adding the global ratings).

### Data analysis

Quantitative data were analysed using descriptive statistics performed using IBM SPSS, version 19. Qualitative data from focus groups and reflective assignment excerpts were analysed using specific types of content analysis.

Focus group data remained grouped under the six questions that were discussed during the session and responses were analysed using Summative Content Analysis. This involves subjective interpretation of the content of text data through systematic classification, coding and identifying themes or patterns [57]. It goes beyond the manifest (or visible) content analysis process of counting frequency of appearance of different words within responses [57], to examining language and its meaning. The researchers (AS, SL and JL) undertook this analysis independently by reading and re-reading responses to each focus group question, word by word, then undertaking memo-writing to begin formulating general impressions about students’ responses. They highlighted words and phrases with similarities and differences in perspectives to identify tentative patterns. They then compared and contrasted students’ responses and scrutinised the memos before finalising the dominant themes and student quotes to demonstrate those ideas within each question.

Excerpts from reflective assignments consisted of responses to the assignment question; ‘What could you have done differently’. Written excerpts were reviewed and analysed using a Transformative Learning Theory concepts and a framework to classify reflective practices as content, process or premise reflections. Mezirow [58] refers to meaning schemes in his Transformative Learning Theory, describing the way individuals perceive events influences what they see and how they see it. Kitchenham [59] provides examples of how reflection practices lead to the transformation of meaning schemes. Simply self-examining actions, referred to as content reflection, has the potential to transform individual meaning schemes. In the context of motivational interviewing this might involve students asking themselves ‘What motivational interviewing skills did I use and why?’ Process reflection involves the consideration of actions as-well as related influential factors. For example, ‘What client and environmental factors were influential in the motivational interview.’ Premise reflection occurs when students achieve a broader perspective and consider a range of influential factors on process and outcome. For example, a student might ask ‘Why is motivational interviewing influential to my clinical practice as well as client outcomes?’

### Ethics

The study received ethics approval from the Flinders University Social and Behavioural Research Ethics Committee.

## Results

All 36 students submitted their written interview transcripts and assignments which described their use of the MITI tool for structured reflection. Although all students were encouraged to report their MITI scores, twenty-two (*n* = 22, 15 female, 13 PT) specifically reported all of their results. A convenient sample of eleven assignments, that were submitted electronically, provided text for content analysis in response to the ‘what would you do differently’ question.

### Focus group

When asked to comment on what made it easier or harder to conduct MI, and why, students made the following comments:

*Awareness of MI complexities*

*It took time to learn about the MITI tool and perhaps a simplified version would have made it easier, 29 pages is a lot to go through. (PT1, male)*

*Transcribing was hardest to do, although it made me much more aware of the interview. (PT2, female)*

*I learnt from the interview and would now ask different questions. (OT1, female)*

*Found it easier to subjectively rate the interview than to quantify the different elements. For example, it was difficult to determine what simple reflection is and what complex reflection is. Also, evocation was found to be confusing. (PT2, female)*

*Learning may be improved by guidance and staging the progress. For example, interview and transcript, identify the elements and rate by using an example of an interview. An example of a motivational interview that is rated in class would have been helpful since scoring is hard. (PT3, female)*

Students shared their perceptions on how the persons they interviewed responded to their questions.

*Good open-ended questions tended to produce useful answers. (PT2, female)*

*Yes, and you could feel there was then more cooperation. (OT1, female)*

Students were challenged when asked about their confidence about the interview process.

*The challenge is not to think of the next question and worrying about the process that you need to follow, and therefore not listening. (PT1, male)*

*Being hung up on goal setting also hampered the interview. (PT3, female)*

*Conducting the interview made me more conscious of using personal preferences of the patient and goal setting. (OT1, female)*

*Knowing that the interview needed to be transcribed influenced the process. (PT4, male)*

*Theory to practice*

In connecting theory and practice, and whether conducting the interview felt like the theory that had been taught, students mentioned:

*The sample questions, for example, about the perceived impact of the problem [on the interviewee] were very helpful and formed a useful starting point, although you then need to let go once the interview starts and rolls along otherwise you don’t listen to what the patient says. (PT2, female)*

*I found decisional balance list on advantages and disadvantage for changing behaviour very helpful. (PT3, female)*

*Directive-task orientation tendencies*

When asked about students’ learning needs to improve their MI skills, there was a general consensus about the need to practice to become more competent. One student commented on the need to focus on the individual.

*I think it is very important to focus on the specific issues of the patient, but this is easy to forget during the interview since we tend to give advice. (PT4, male)*

*Clinical relevance of MI*

When asked if they thought motivational interviewing was applicable to their future clinical practice, there was also general consensus that it was important.

*MI is useful but I would prefer to use elements of it in future practice as a kind of integrated approach. For example, identifying barriers and enablers in chronic disease management, individual preferences or negotiating goal setting. (PT1, female)*

### Assignment excerpts

When responding in their assignments to ‘What could you have done differently’ students demonstrated reflection on their interview performance and their learning experiences. The text of the excerpts is in line with the results of the focus group session on the elements of the interview that generally need attention and how these could be improved. Table 1 shows the types of reflection for indicators of transformation, defined as content, process or premise in Mezirow’s Transformative Learning Theory [60].

**Table 1:** Types of reflection of students as indicators of transformation

Reflection	Definition	Example
Content	Learning with present meaning by thinking back to what was done	I found some principles such as empathy and supporting autonomy came almost intuitively while other concepts like open ended questions and complex reflections proved to be more difficult to adopt. (PT7, female)
		Overall the interview-reflection experience provided me with an opportunity to apply theoretical principles of motivational interviewing and then reflect on the process (OT4, female)
		During the interview I gave my client the responsibility to discover different strategies he could use to improve his motivation and also the tools and strengths he already had that he should emphasize. (PT7, female)
Process	Learning with new meaning by considering actions and related factors	Equally, I need to work on my questions to make them more reflective and open-ended. (OT7, female)
		As a next step, I will focus on restructuring the interview in a way which will benefit power sharing, collaboration and self-efficacy especially during goal setting and working through the decisional balance sheet. (OT2, male)
		Instead, due to inexperience, I feel as though I was too focussed on planning what I could say next, rather than actively listening to her responses and incorporating them in my interview (OT6, female)
		Throughout the MI I should have sought to evoke his ‘change talk’ ………… and I could have responded better with reflective listening (PT1, male)
		I would make a mindful effort to the clients control and highlight their power within their ability to decide their goals and course of action. (OT8, female)
		If I were to perform this task again I would make a concerted effort to probe further into the patient’s responses and reasoning. This would promote a greater understanding of the patients health barriers and barriers to change and ideally would result in more detailed goals, more structured health plans and better outcomes. (PT5, male)
		I believe that I made it clear to my client that he was free to make his own choices, I wasn’t there to dictate to him, and he was required to self-direct in order to discover answers and ways to change his behaviour. (PT7, female)
		Considering this interviewer did not meet the beginner’s threshold proficiencies (Moyers et al. 2007), it’s worthwhile concluding by reflecting on how skills in motivational interviewing can best be developed ….. (OT5, female)
Premise	Learning through meaning transformation by considering a broader perspective	Providing reflective responses to interviewee’s statements would allow not only greater collaboration and direction but also allow the interviewee to walk away feeling more empowered. (OT2, male)
		To further develop client discrepancy, it is important why change may not be a good idea, or consequences of not changing in addition to why change is a positive factor. (OT4, female)
		It is vital that the interview support the client in their decision and to ensure that questions are not being repeated as this can cause resistance and / or friction with the client (OT3, female)
		It has educated me that people are often ambivalent about health behaviours and that resistance and change are two sides of a coin (PT1, male)
		While motivational interviewing is difficult to master, it is a very important skill to enable effective communication between a practitioner and client. This is a skill that I intend to practice for my career as an Occupational Therapist (OT6, female)

### MITI scores

Global Spirit scores ranged between 2.67 and 4.67 (*M* = 3.73, SD .5), which is mid way between the proficiency classification of beginner and competent [52]. The students were new to MI and according to Moyers and colleagues [52] beginner proficiency is classified by a Global-Rating Score 3. Our sample self-rated themselves with scores of between 3 and 4, producing a mean score of 4. No gender differences in means scores were detected, although the male sample (*n* = 7) was smaller than the female sample (*n* = 15). Five sub-scores with a scale between one and five measure the extent to which the clinician perceive they demonstrate the defined MI behaviours. As shown in Table 2, students self reported high level MI skills with a mode of four for all sub-scores (evocation, collaboration, autonomy, direction and empathy).

**Table 2:** Perceived MI proficiency per item and for the global spirit of the interview

Item	Minimum	Maximum	Mode	Frequencies of scores in %			
				2	3	4	5
Evocation	3	5	4	0.0	22.7	59.1	18.2
Collaboration	2	4	4	4.5	40.9	54.5	0.0
Autonomy	2	5	4	4.5	27.3	59.1	9.1
Direction	3	5	4	0.0	9.1	59.1	31.8
Empathy	3	5	4	0.0	40.9	54.5	4.5
			Mean	SD	IQR		
					25	50	75
Global Spirit Score	2.67	4.67	3.73	.50	3.33	3.67	4.00

## Discussion

This pilot study indicated that a self-reflection of MI using the MITI tool challenged the students and brought about what appears to be a transformative learning outcome. Students articulated a tension between being goal-focused as opposed to working and being with the ‘client’ whilst interviewing. There was general agreement that the self-assessment-reflection activity made them aware of what MI is, processes that needs to be followed, some of the barriers and enablers that can be encountered, and how they can improve their performance.

This research focused upon the process and outcome of teaching pre-registration health students. Previous work has considered aspects of teaching MI to experienced and qualified clinicians [61–63]. This study builds upon this work and considers the process and learning outcomes of reflective writing in conjunction with the MITI tool to train students who are not experienced working as health practitioners. A further distinction is that we trialled students rating their own MI performance rather than supervisors scoring the students which is the traditional practice [64, 65].

Students were expected to complete multiple elements. The practice method of mock interviews has been found to result in greater MI learning outcomes when compared with written work [7]. Our sample reported benefits of both, and future research might consider alternate combinations of learning tasks and outcomes based upon student experience and confidence levels. Similarly, interviews involving clients unknown to the students may generate different challenges and opportunities for high fidelity MI training.

Our findings indicate that the MITI tool, apart from being a tool for supervisors, is also useful for students to review their own MI work to achieve greater self awareness. The structure of the MITI also provides a framework for on-going self-reflection and development as the student transitions into the workforce.

MI training is already incorporated into a wide range of undergraduate and graduate-entry programs. However, there are challenges to effectively instilling skills to foster continued use after transition to the health workforce. Literature supports the benefits of students maintaining MI skills through practice, to enhance the impact of clinical practice in relation to health outcomes [66]. However, MI skills are difficult to acquire and tend to decay over time, training needs to be accompanied by supervised interviewing and the provision of feedback [22], to enhance and maintain the quality of these skills [1]. Teaching MI, therefore, can be resource-intensive, with learning outcomes that may be superficial and also difficult to further develop and maintain once students leave the learning environment.

Since the MITI tool allows validated reflection on performance, its use can encourage students to become reflective practitioners which may enable them to maintain their interviewing skills during clinical placement and after graduation, as part of lifelong learning [67, 68]. In answering the second research question, we found that students reported higher than expected MITI scores. One explanation for this is that post-graduate health students who are yet to enter the workforce may over-rate their MI skills. So even though the self-reflection activity promotes improved awareness of the complexities of MI and transformative learning, it may be advantageous to introduce a peer review process that compliments the self-assessment. Future research may explore the self-rated scores of students in a work-place setting and whether they alter with exposure to real-life MI scenarios.

Assessment has the potential to increase the depth of learning [35]. The purpose of assessed reflective work during training is to prepare students for reflective practice [36, 37]. Although students were given guidance on the reflective assignment (Additional file 1) and assessment criteria (Additional file 2), ownership of the criteria could have been enhanced by development of an agreed marking matrix in class. Also, it is not certain that students will apply reflective practice after graduation, or whether they become more effective over time or will revert back to offering solutions not owned by their clients.

It is possible that, in line with findings about nurses [51], practitioners’ task-orientation (i.e., focusing on treating the disorder) may be a barrier to recognising factors that could compromise motivation and program adherence of clients. Although the purpose of conducting a focus group session was primarily to find out how students perceived this approach to learning MI, the focus group results showed that task-orientation can be an issue for some students. If so, then preparing health students adequately for chronic condition management has consequences for university programs, particularly for students in the more technical or more task-orientated professions where practitioners are used to telling their clients the solutions instead of negotiating decision-making about lifestyle choices.

### Limitations

A possible limitation of this study is the use of family and friends as subjects, rather than real clients or trained simulated clients. The former group has the potential to collude with the interviewer and readily alter their motivation in response to student’s efforts. Their ‘stories’ may be more artificial or possibly staged. However, as mentioned earlier, the aim was not to rate students on their actual interview, but for students to reflect on their self-assessed MI performance required them to take note of what constitutes a good interview. To improve actual interview performance, future studies could consider using real or trained simulated clients to mirror the clinical reality of behaviour change.

Although a 2010 version of the MITI tool exists, reproduction restrictions apply that limit accessibility for educators and students. The 2007 version [52] is co-authored by William Miller and in line with what students were taught, and was the preferred tool.

A potential limitation is that students were not required to submit a logbook to record coding practice. Although students were encouraged to practice and follow the recommendations of Moyers and colleagues [52], to start coding Level I competencies before coding Level II and Level III competencies, there was no requirement for them to keep a log to show that they did this or practiced for the recommended amount of hours (Moyers and colleagues recommend 40 h of practice); hence, they may not have had sufficient exposure to the tool to develop the skills to rate their performance.

Another limitation is that students may have felt that they needed to participate in the project since the principal researcher is also the topic coordinator. Students were clearly informed that participation was voluntary, although writing a reflective assignment was an expectation. Also, non-participation was not going to affect their mark in any way, and low focus group attendance illustrates that students were not coerced into participation. The focus group session was conducted after the assignments were marked. The focus group date coincided with most OT students being on clinical placement, which impacted on their availability to attend. This problem was mitigated by making the questions available to all students prior to the focus group so they could send a representative. Although the questions may have been fairly direct, the idea was to receive succinct and tangible feedback that could be used to improve the delivery of this topic.

This study did not quantify students’ self-rated performance, nor did it validate their performance against that of MI experts. The focus was on students submitting a quality reflective assignment rather than a quality MI interview in which they could demonstrate effective use of MI skills. This standpoint was taken because it was the quality of their reflection that was deemed important, also given that they were mostly novices and may have felt pressured if rated on their interview. Despite this provision and emphasis, some students appeared to submit transcripts in which their interactions seemed staged, to possibly show effective use of MI skills. Also, it needs to be determined whether interviewing a family member or friend is an enabler or barrier to learning to conduct a MI. If it is not found to be a barrier then, except for the possibility of exposing students to a virtual training environment, this approach is a first step and likely to require fewer resources than using real clients or simulation that utilises actors. Although students interviewing people known to them, and therefore more likely having an underlying positive relationship with them prior to the MI, may have contributed to the higher rating in the MITI score, the next step could be to then interview a real client once they have a greater sense of mastery.

## Conclusion

This study provides a strategy that seems effective in recognising the MI processes and facilitating the acquisition of MI skills in health students by reflecting on self-assessment. Although some students found it challenging, findings of this study indicate that it was a deep transformative learning experience that may inform teaching programs across the different health professions about how to best teach MI skills to health students and what methods are more effective in helping them to acquire these complex skills.

### Practice implications

Traditionally, MI is taught by receiving one or several presentations, followed by watching an expert and some time to practice under supervision with a peer. The strength of this study was its interprofessional approach to teaching MI to students from different disciplines, and preparing them for reflective practice. Given that universities have to be increasingly resourceful and creative in teaching health students to become competent practitioners, reflective self-assessment against validated tools appears to be a way to achieve this.

## Additional files

Additional file 1:
**Reflective assignment – Process, content and structure.** (DOCX 21 kb) Additional file 2:
**Reflective Assignment – Detailed Assessment Criteria.** (DOCX 21 kb)
