# Supplementary material for: Teaching health science students foundation motivational interviewing skills: use of motivational interviewing treatment integrity and self-reflection to approach transformative learning
Source: BMC Med Educ. 2015 Dec 21;15:228. doi: 10.1186/s12909-015-0512-1 (PMC4687369; doi:10.1186/s12909-015-0512-1)
Supplement: Additional file 1: — Reflective assignment – Process, content and structure. (DOCX 21 kb) [file 12909_2015_512_MOESM1_ESM.docx]

**Additional file 1: Reflective assignment – Process, content and structure**

1. Determine the topic of the interview (usually as agreed with the patient, but for the purpose of this assignment it will be on physical activity or exercise, or the use of a device to aid function/recovery).
2. Prepare the interview.
3. Conduct the interview, and record it. An information sheet and consent form will be uploaded on FLO so that your ‘patient’ can provide informed consent.
4. Transcribe the interview.
5. Reflect on the actual interview. This can include, but is not limited to:

- What are the barriers of your ‘patient’?
- What are the enablers of your ‘patient’?
- Do you recognise some of the issues that have been covered earlier in the topic, and what were they?
- Did you feel resistance or cooperation and, if so, when?
- Were you able to facilitate some goals?
- Before you compare your interview against the MITI tool, how do you feel it went?

In any of the points mentioned above you can refer to the literature as appropriate (e.g., rurality, communication/counselling and or goal setting).

6. Appraise your interview and validate against the MITI tool. This can include, but is not limited to:

- Rate your performance on each of the items and calculate your global ratings and behaviour counts.

- Do the results surprise you?

- What did you do well in the interview, and why?

- What did you not do so well, and why?

- What have you learnt from this?

- What would you do differently, and why?

In any of the points mentioned above you can refer to the literature on motivational interviewing as appropriate.

7. Submit your assignment together with recording and transcript of the interview, and the signed consent form.

**Recommended reading**

- Epstein, R. (1999). Mindful practice. The Journal of the American Medical Association, 282(9), 833-839.
- Taylor, B.J. (2010). Reflective practice for healthcare professionals : A practical guide 3rd ed. Maidenhead ; New York : Open University Press.
- Kember, D., McKay, J., Sinclair, K., & Wong, F.K.Y. (2008). A four-category scheme for coding and assessing the level of reflection in written work. Assessment & Evaluation in Higher Education, 33(4), 369-379.
- Moon, J.A. (2004). *A handbook of reflective and experiential learning: Theory and practice.* New York: RoutledgeFalmer.

**Success!**
