# Supplementary material for: Teaching health science students foundation motivational interviewing skills: use of motivational interviewing treatment integrity and self-reflection to approach transformative learning
Source: BMC Med Educ. 2015 Dec 21;15:228. doi: 10.1186/s12909-015-0512-1 (PMC4687369; doi:10.1186/s12909-015-0512-1)
Supplement: Additional file 2: — Reflective Assignment – Detailed Assessment Criteria. (DOCX 21 kb) [file 12909_2015_512_MOESM2_ESM.docx]

**Additional file 2: Reflective Assignment – Detailed Assessment Criteria**

| **Rating** | **Understanding**  **(20%)** | **Synthesis**  **(20%)** | **Evaluation (20%)** | **Reflection**  **(30%)** | **Presentation**  **(10%)** |
| --- | --- | --- | --- | --- | --- |
| High Distinction [Outstanding Work]  85 – 100 | Exceptional knowledge and in-depth understanding Comprehensive discussion of relevance to clinical and interprofessional practice | Exceptional ability to select & apply relevant material.  Outstanding use of explanation and summarisation | Critical insightful evaluation of issues and material with an original and reflective approach. Extensive use of evidence | Exceptional ability to reflect using wide range of relevant examples | Professional writing style throughout with appropriate grammar and spelling  Accurate referencing |
| Distinction [Excellent work]  75 - 84 | Excellent knowledge and depth of understanding  Discusses relevance to clinical practice and interprofessional working | Excellent ability to select & apply relevant material  Excellent use of explanation and summarisation | Effective evaluation and critical analysis of main issues and some minor issues with reference to key evidence | Excellent ability to reflect using many relevant examples | Written style clear and effective most of the time. Consistent use of standard grammar and punctuation. Very occasional referencing errors |
| Credit  [Very good work]  65 - 74 | Comprehensive knowledge and depth of understanding  Describes the main applications to clinical and interprofessional working | Sound ability to select and apply  relevant material  Evidence of explanation & summarisation | Critical evaluation of most major issues  Draws upon varied range of evidence to support reflection | Good ability to reflect using a range of examples | Sentence constructions generally correct.  Occasional spelling and grammar errors.  Minor referencing errors |
| Pass [Satisfactory work]  50 – 64 | Appropriate knowledge and understanding  Some reference to clinical practice and interprofessional working | Relevant application of materials with some minor omissions  Good attempt to explain and sum up | Accurate description of main issues with some critical evaluation. Some use of relevant research to support reflection | Attempt to reflect with some examples. | Some problems with sentence construction. Written style wordy or repetitive at times. Some spelling and grammar and referencing errors |
| Fail  [Limited work]  40- 49 | Limited knowledge and some omissions  Limited emphasis on applications | Limited evidence of relevant application  Limited attempt to explain & sum up | Limited critical analysis  Literature is presented descriptively | Limited evidence of reflection | Frequent problems with sentence construction. Inappropriate word choice. Many spelling mistakes and referencing errors |
| Fail [Unacceptable] 0 – 39 | Insufficient, largely irrelevant information | Insufficient evidence of application | No evidence of critical analysis. Literature not consulted or irrelevant | No evidence of reflection | Unacceptable writing style with numerous errors. Inadequate referencing |
